# Evolution of the Strengthening Effects of In Situ TiB Fibers in a Ti Composite with Various Levels of Thermal Processing

**DOI:** 10.3390/ma16062472

**Published:** 2023-03-20

**Authors:** Peifeng Zhao, Fengcang Ma, Ping Liu, Wei Li, Xinkuan Liu, Xiaohong Chen, Ke Zhang

**Affiliations:** 1 School of Materials Science and Engineering, Henan University of Science and Technology, Luoyang 471000, China; 2 School of Materials and Chemistry, University of Shanghai for Science and Technology, Shanghai 200093, China

**Keywords:** metal matrix composites (MMCs), fiber orientation, fiber aspect ratio, strengthening effect, thermal processing route

## Abstract

Thermal processing is a useful method for improving the strengthening effects of fibers used to reinforce metal matrix composites (MMCs), but the corresponding models have not been constructed. In this work, a Ti matrix composite (TMC) reinforced by in situ TiB fibers was prepared, then thermal processing was applied to it at different levels of deformation to align the TiB fibers along the loading direction. Changes in the microstructure of the matrix, the orientation and the aspect ratio of the TiB fibers during this process were investigated. It was found that the aspect ratio of TiB fibers decreased sharply after a large amount of deformation. The strengthening effect of TiB fibers in the composite was simulated by strengthening models of the fibers, and the simulated results were verified by the results of tensile tests. The modeled results show that the strengthening factor (*C*_0_) of the in situ TiB fibers improved from 0.125 in the as-cast composite to 0.520, 0.688 and 0.858 by the processes with deformation ratios of 0.39, 0.26 and 0.14, respectively. The results of the tensile tests showed that the measured values of C_0_ gradually deviated from the modeled ones with an increase in the ratio of deformation applied during processing, and the deviation could be interpreted with the strengthening models.

## 1. Introduction

Because of their high elastic modulus and strength, fibers (carbon, carbides, oxides, borides and nitrides) are widely used in composites such as metal and polymer matrix composites [1,2,3,4,5]. Ceramic or carbon fibers added into a composite can increase its matrix strength, elastic modulus and service temperature [6,7,8,9]. Over the past few decades, interest in the structural use of metal matrix composites (MMCs) in the aerospace, automotive and other industries has increased with the development of various low-cost methods of preparing fiber reinforcements and MMCs. The use of ceramic fiber-reinforced metal has increased rapidly, as it offers a combination of good mechanical properties and a low preparation cost [10,11,12].

Fiber reinforcements added into MMCs include continuous long fibers and discontinuous short fibers. Compared with MMCs reinforced with long fibers, MMCs reinforced with short fibers have many advantages, such as their low preparation cost and easy fabrication, and the availability of pressure processes used for metals [13,14,15]. Among the many methods of preparing MMCs reinforced with short fibers, the in situ synthesis reinforcement technique, in which the reinforcement fibers form in situ in the matrix through the reaction between the matrix and the reactant, has been paid more attention because of the better compatibility of the matrix with the reinforcement fibers and the lower contamination of the interface compared with the ex situ technique [16,17,18,19].

In recent years, traditional pressure processes for metals such as forging, rolling and extrusion have been applied to MMCs in many studies. These studies have shown that the mechanical behavior of MMCs is affected by the fibers’ orientation, which can be controlled by the processing method applied to the MMCs [13,16,20,21,22]. As reported by Gorsse et al. [23], when the orientation of the fibers’ length is parallel to the loading direction, the composite has the highest strength. Another study also showed that pultruded thermoplastics reinforced with continuous glass fibers had higher axial stiffness and strength than other loading directions [24]. For a composite reinforced with short fibers, many processing methods are available to change the fibers’ orientation in the final composite and hence its mechanical properties.

A number of studies have been carried out on the techniques used to produce the preferred fiber orientation in composites reinforced with short fibers. These techniques include rolling, drawing, extruding and other methods of deformation [13,16,25,26,27,28]. In the research of Guo et al. and Ma et al. [29,30,31,32,33], the fibers’ orientation and its evolution during rolling was investigated, and a planar model was constructed to predict the distribution of the fibers’ orientation and the changes after rolling with different deformation ratios. This model of the change in the fibers’ orientation was used by the authors to calculate the strengthening effect of the TiB fibers, and the calculated values agreed well with the experimental results [32,33]. However, this model can only be used in planar deformation processes such as rolling and compressing. In practice, three-dimensional deformation processes are used more widely because of their greater efficiency compared with planar deformation processes. However, the evolution of the fibers’ orientation and the changes in the mechanical properties after these processes are more complex than those of planar deformation processes. To the best of our knowledge, related models on this subject have not been constructed.

In this study, a three-dimensional deformation process was applied to a composite reinforced with short fibers to produce the preferred fiber orientation in the composite. The changes in the fibers’ orientation and the strength of the composite with different fiber orientations achieved by this process were modeled.

## 2. Experimental Procedures

### 2.1. Preparation of the Composite

**Figure 1:**
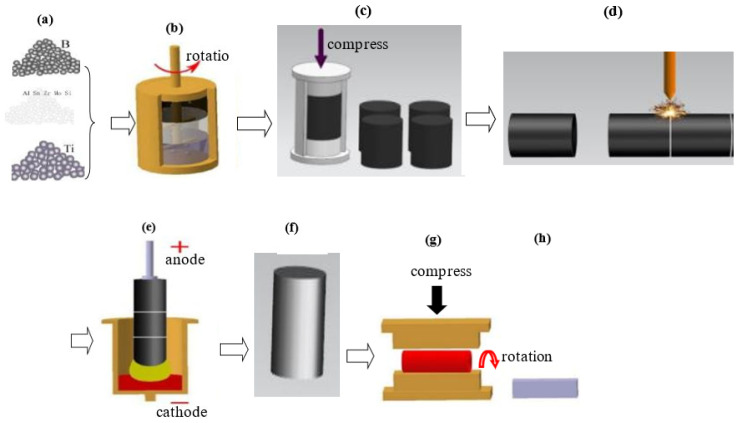
Schematic of the preparation and thermal deformation of the composite reinforced with short in situ TiB fibers. (**a**) raw powders; (**b**) mixed mechanically; (**c**) pressed into rod; (**d**) rods were welded as an electrode; (**e**) electrode were remelted in a vacuum arc remelting furnace; (**f**) composite casting; (**g**) composite performed thermal-processing; (**h**) composite after thermal-processing.

The composite prepared in this study was 4 vol.% TiB-reinforced Ti–6Al–2.75Sn–4.0Zr–0.4Mo–0.45Si alloy. Figure 1 illustrates the preparation and thermal deformation of the composite. In the preparation of this composite, titanium sponges (Grade I), aluminum threads (98% purity), zirconium sponges (99.5% purity), silicon powder (99.95% purity, a particle size of 6–12 µm), and Al–Mo and Ti–Sn master alloys were used as the raw alloy materials. Boron (purity 99.5%, particle size 2~4 µm) was added as the reactant to form TiB reinforcement, and the nominal volume fraction of reinforcement was 4 vol.% calculated with Equation (1).

(1) $$i_{V}=\frac{\frac{i_{M}}{\rho_{R}}}{\frac{i_{M}}{\rho_{R}}+\frac{1-i_{M}}{\rho_{M}}}$$

where $i_{V}$, $i_{M}$ and $\rho_{R}$ are the volume fraction, mass fraction and density of TiB, respectively; $\rho_{M}$ is the density of titanium.

**Figure 2:**
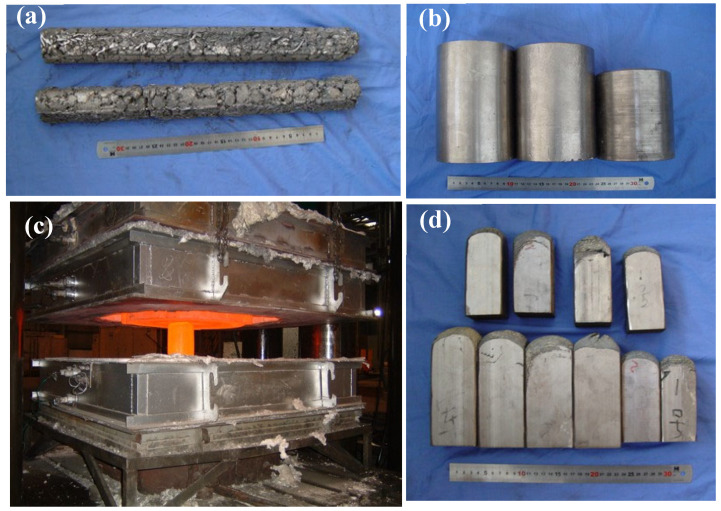
Photographs of the sample during the preparation of the composite. (**a**) Electrode made of the mixture of raw materials. (**b**) Ingots of the composite. (**c**) Thermal deformation of the composite. (**d**) The composite after thermal deformation.

First, stoichiometric raw alloys and boron powder were mixed mechanically. Second, the mixture of raw materials and the reactant were pressed into rods with a die on a hydraulic press (Figure 2a, Figure 1c), and these rods were welded as an electrode to be used for remelting. Next, the electrodes were remelted in a vacuum arc remelting furnace. During the process of remelting the electrode, TiB was synthesized by the reaction Ti + B→TiB. The ingots of the composite (Figure 2b, Figure 1f) were remelted twice to improve chemical homogeneity, and thermal deformation of composite ingots was performed with a horizontal hydraulic press (Figure 2c, Figure 1g). Figure 2 shows photographs of the sample during preparation of the composite. Finally, a homogenization treatment was carried out on the ingot of the composite at 1423 K for 1.5 h, then it was air-cooled to ambient temperature. The ingot of the composite was machined to remove surface defects (Figure 2d, Figure 1h).

### 2.2. Thermal Deformation

Thermal deformation of the composite was performed with a horizontal hydraulic press equipped with electrically heated upper and lower molds. Before thermomechanical processing, the ingot of the composite was heated to 1323 K in a furnace; meanwhile, the dies used in the open-die forge were heated electrically to 1173 K. During thermal deformation, the temperature of the composite was monitored with an infrared radiation thermometer to prevent cracks forming from lower temperatures at the end of forging. After thermomechanical processing, the composite rods were air-cooled to ambient temperature. The deformation ratio was used to characterize the extent of deformation, and it was calculated as $\lambda =\frac{l_{0}}{l_{i}}$, where $l_{0}$ and $l_{i}$ are the length of the composite sample before and after thermomechanical processing, respectively. The thickness of the sample during thermomechanical processing was measured and controlled by a meter fitted to the hydraulic presser. The same extent of deformation along the X direction and the Y direction in the cross-section of the ingot was applied during this process. The thermal deformation process is illustrated in Figure 1.

### 2.3. Characterization of Microstructure and Mechanical Properties

Metallographic samples were cut from the composite for observation with an optical microscope before and after thermomechanical processing. These were polished mechanically and etched with Kroll’s reagent (3–6 mL HF and 5–10 mL HNO_3_ per 100 mL water). Scanning electron microscopy observations were carried out on FEG Quanta 450 equipment. The foils used in the transmission electron microscopy (TEM) analysis were prepared with a twin-jet device working at 30–50 V at a temperature range of 293–303 K, and the electrolyte was 10% HClO_4_ and 90% CH_3_COOH. TEM analysis was carried out on a JEM-2010 microscope working at 200 kV.

Tensile cylindrical specimens were cut and machined with their central axes parallel to those of the composites before or after thermal-mechanical processing. These cylindrical specimens were designed according to ISO 783: 1999(E). The gauge size of the tensile cylindrical specimens was 50.0 mm and 10.0 mm in length and diameter, respectively, and the size of the specimen holders was 25.0 mm and 20 mm in length and diameter, respectively. The tensile behavior of the composite was tested on a Zwick/Roell 50 kN tensile testing machine. Before the tensile test, the sample was heated to the test temperature in a furnace on this tensile machine. The strain rate used in these tensile tests was 5.0 × 10^−3^ s^−1^. To avoid random results, the mechanical properties of each composite were tested three times and the presented results were the averages of the three values.

## 3. Results and Discussion

### 3.1. The Morphology and Formation Mechanism of TiB Fibers

**Figure 3:**
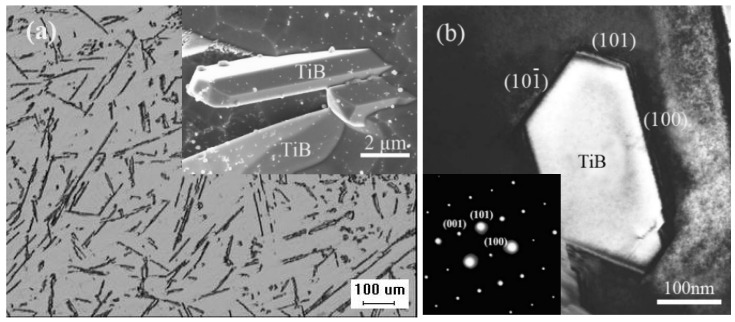
(**a**) Optical morphology and scanning electron microscopy images of the in situ TiB fibers. (**b**) Transmission electron microscopy (TEM) and selected area diffraction patterns of in situ TiB fibers in the as-cast composite.

Figure 3a shows the morphology of the in situ TiB fibers after deep etching and their distribution in the as-cast composite. As shown in Figure 3a, the diameter of the in situ TiB fibers formed by arc remelting and casting was ~2 μm with a high aspect ratio. The in situ TiB fibers had a needle morphology and the surface was flat and clean. Figure 3b shows the bright field TEM images of the TiB and corresponding diffraction patterns of the selected area. Previous research showed that needle-like TiB fibers have a B27 orthorhombic crystal structure with a = 0.612 nm, b = 0.306 nm and c = 0.456 nm [34].

The morphology of in situ TiB fibers may result from their crystal structure and growth mechanism. Lundstrom [35] reported that the crystal structures of many metal borides have a similar crystal cell in which a boron atom is at the center of a trigonal prism, while the six vertices of the trigonal prism are occupied by Ti atoms. The adjacent trigonal prisms are connected by their shared edges. A zigzagging chain is formed by boron atoms along the [010] crystal direction of the B27 structure, and Ti atoms form boron-free ‘pipes’ with a trapezoidal cross-section. As suggested in the periodic bond chain model of crystals, TiB tends to grow along the [010] crystal direction into fibers, and its axis is parallel to the [010] crystal direction of B27 [36].

### 3.2. Changes in Microstructure during Thermal Deformation

#### 3.2.1. Changes in the Orientation of TiB Fibers

**Figure 4:**
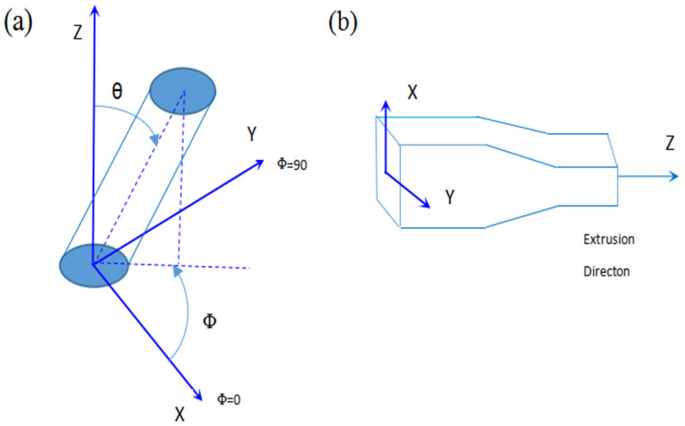
(**a**) Schematics of the TiB fibers’ orientation in the composite. (**b**) Changes in the TiB fibers’ orientation during thermal deformation.

Figure 4a shows the changes in the TiB fibers’ orientation during thermal deformation. As seen in Figure 4b, according to the pseudo-affine deformation scheme, the orientation angles θ and Φ of the fibers after a general compression deformation are related to the original orientation angles θ′, Φ′ and the deformation ratio. The deformation ratio can be defined by λx, λy and λz, which indicate the deformation ratio along the X, Y and Z axes, respectively. Changes in the TiB fibers’ orientation caused by thermal deformation can be predicted by the following two equations [37].

(2) $$\tan\theta =\frac{\lambda X}{\lambda Z}\tan\theta^{\prime }{\left[\frac{\left(1+{\left(\frac{\lambda Y}{\lambda X}\right)}^{2}\tan^{2}\varphi^{\prime }\right)}{\left(1+\tan^{2}\varphi^{\prime }\right)}\right]}^{\frac{1}{2}}$$

(3) $$\tan\varphi =\frac{\lambda Y}{\lambda X}\tan\theta^{\prime }$$

Because of the constant volume of the composite sample during deformation (i.e.,λXλYλZ=1), for the compression deformation performed along the X direction, the width of the sample was approximately constant so that λ_Y_ = 1 and λ_X_ = l/(λ_Z_). In this case, Equations (2) and (3) are reduced to

(4) $$\tan\theta =\frac{1}{\lambda^{2}}\tan\theta^{\prime }{\left[\frac{\left(1+\lambda^{2}\tan^{2}\varphi^{\prime }\right)}{\left(1+\tan^{2}\varphi^{\prime }\right)}\right]}^{\frac{1}{2}}$$

(5) $$\tan\varphi =\lambda \tan\varphi^{\prime }$$

where λ = λ_Z_. In this work, because the same extent of deformation was applied in the X and Y directions, then

$$\lambda_{X}=\lambda_{Y}=\frac{1}{\sqrt{\lambda_{Z}}}$$

Equations (2) and (3) can then be simplified to

(6) $$\tan\theta =\lambda^{\frac{3}{2}}\tan\theta^{\prime }$$

(7) $$\varphi =\varphi^{\prime }$$

These equations are identical to the results of a number of other studies that aimed to resolve purely elongational deformation [38,39]. In Figure 4b, $P\left(\theta^{\prime }\right)$ and P(θ) are the probability density functions of the TiB fibers’ orientation before and after thermal processing, respectively.

(8) $$P\left(\theta \right)d\theta =P\left(\theta^{\prime }\right)d\theta^{\prime }$$

Because the same extent of deformation was applied along the X direction and Y direction in this work, Equation (6) was applicable. Therefore, the orientation angle θ after deformation can be predicted by Equation (9)

(9) $$\theta =\arctan(\lambda^{\frac{3}{2}}\tan\theta^{\prime })$$

Equation (9) can be rewritten for θ as follows:

(10) $$\frac{d\theta }{d\theta^{\prime }}=\frac{tan^{2}\theta^{\prime }+1}{\lambda^{\frac{3}{2}}tan^{2}\theta^{\prime }+\lambda^{-\frac{3}{2}}}$$

If we substitute Equation (6) $\tan\theta =\lambda^{\frac{3}{2}}\tan\theta^{\prime }$ into Equation (10), we obtain

(11) $$\frac{d\theta }{d\theta^{\prime }}=\lambda^{-\frac{3}{2}}sin^{2}\theta +\lambda^{\frac{3}{2}}cos^{2}\theta$$

By substituting Equation (11) into Equation (8), we have

(12) $$p\left(\theta \right)=\frac{p\left(\theta^{\prime }\right)}{\lambda^{-\frac{3}{2}}sin^{2}\theta +\lambda^{\frac{3}{2}}cos^{2}\theta }$$

**Figure 5:**
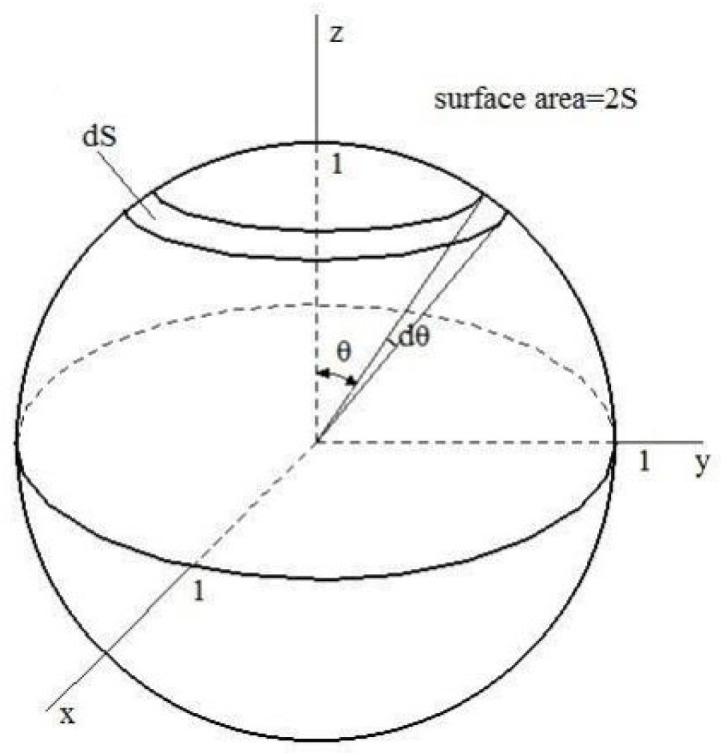
Initial distribution of the orientation of the in situ TiB fibers in the composite.

Before deformation was applied during thermal processing, the TiB fibers’ orientation was distributed randomly in the as-cast composite. A spherical coordinate system was used to characterize the probability density function of this random distribution of the TiB fibers’ orientation, as presented in Figure 5; p(θ)) can be expressed as shown in Equation (13).

(13) $$p\left(\theta^{\prime }\right)d\theta^{\prime }=\mathrm{dS}/S$$

From Equation (13), the following equation can be deduced

(14) $$p\left(\theta^{\prime }\right)=\sin\theta^{\prime }$$

We can substitute $\theta^{\prime }=\arctan(\lambda^{-\frac{3}{2}}\tan\theta )$ from Equation (6) into $p\left(\theta^{\prime }\right)=\sin\theta^{\prime }$ (Equation (13)) and thus obtain

(15) $$p\left(\theta^{\prime }\right)=\sin\left(\mathrm{arctg}\left(\lambda^{-\frac{3}{2}}\tan\theta \right)\right)$$

By combining Equation (15) into Equation (12), we have

(16) $$p\left(\theta \right)=\frac{\sin\left(\arctan\left(\lambda^{-\frac{3}{2}}\tan\theta \right)\right)}{\lambda^{-\frac{3}{2}}\sin^{2}\theta +\lambda^{\frac{3}{2}}\cos^{2}\theta }$$

**Figure 6:**
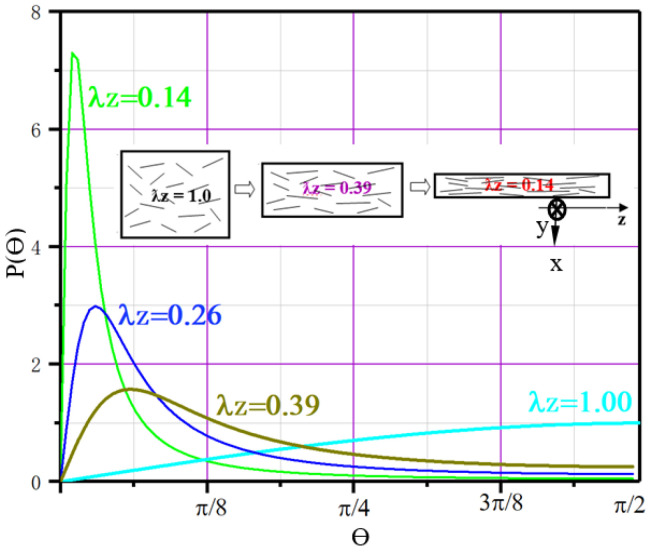
Modeled probability density of the orientation of in situ TiB fibers after thermal deformation with different ratios of deformation.

Equation (16) was used to calculate the $P\left(\theta \right)$ of the in situ TiB fibers with Origin 8.0 software in the casting composite or after thermal deformation with different deformation ratios (λz = 1.0, 0.39, 0.26 and 0.14, respectively); Figure 6 by Origin presents these curves of $P\left(\theta \right)$ after deformations used in this work. As presented in Figure 6, the probability density distribution function shifts to a small angle with increasing deformation amplitude, and its maximum value reached less than π/16 as λz = 0.26 or 0.14, showing significant alignment of TiB fibers by deformation processing.

#### 3.2.2. Changes in the Size of the Alpha Colonies and the Aspect Ratio of TiB Fibers

**Figure 7:**
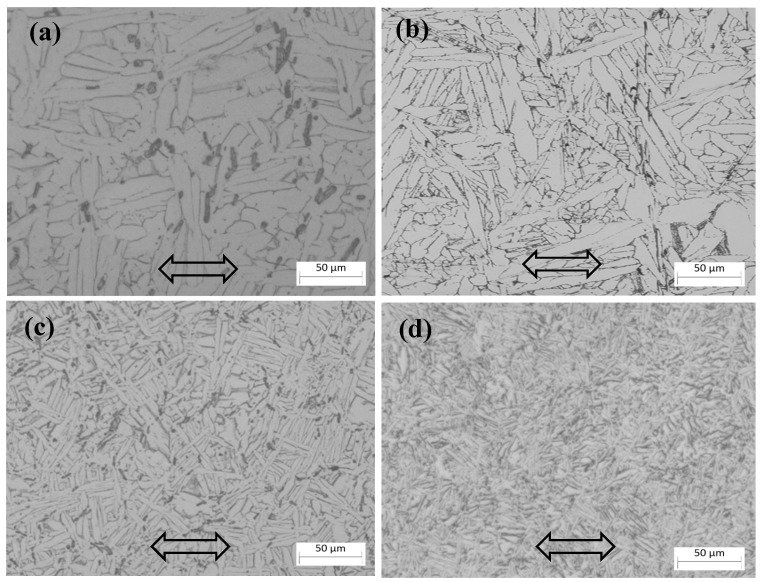
Microstructure of the composite with different deformation ratios, λz: (**a**) 1.0, (**b**) 0.39, (**c**) 0.26, (**d**) 0.14, arrows showing loading direction during tensile tests.

**Figure 8:**
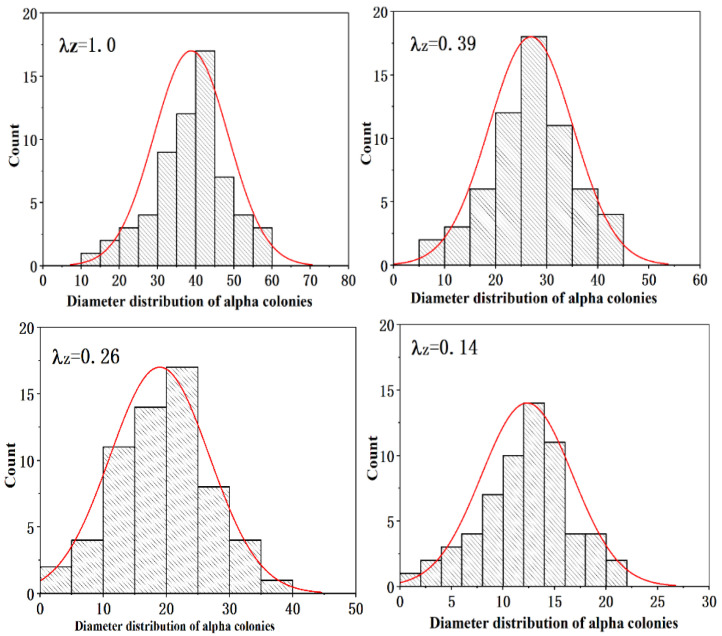
Changes in the distribution of the size of the alpha colonies during thermal deformation.

As shown in Figure 7, the microstructure of this composite before and after thermomechanical processing consisted of multiple alpha lath colonies. The composite’s microstructure became finer after thermomechanical processing. The refinement of the microstructure through this process was achieved by two means. One means of refinement was the dynamic recrystallization of the beta grains. In this case, a larger deformed beta grain turned into several finer new grains through dynamic recrystallization. Refinement could also take place in the transformation process from the beta phase to the alpha phase during the cooling process after thermal processing. Several finer alpha colonies may form in the small recrystallized beta grains, and our previous work showed that the strength of the composite is related to the size of the alpha colonies, to some extent [13,16]. Figure 8 shows the change in the size of the alpha colonies under different deformation ratios. These results indicate that the alpha colonies of the composite were refined significantly by thermal deformation.

**Figure 9:**
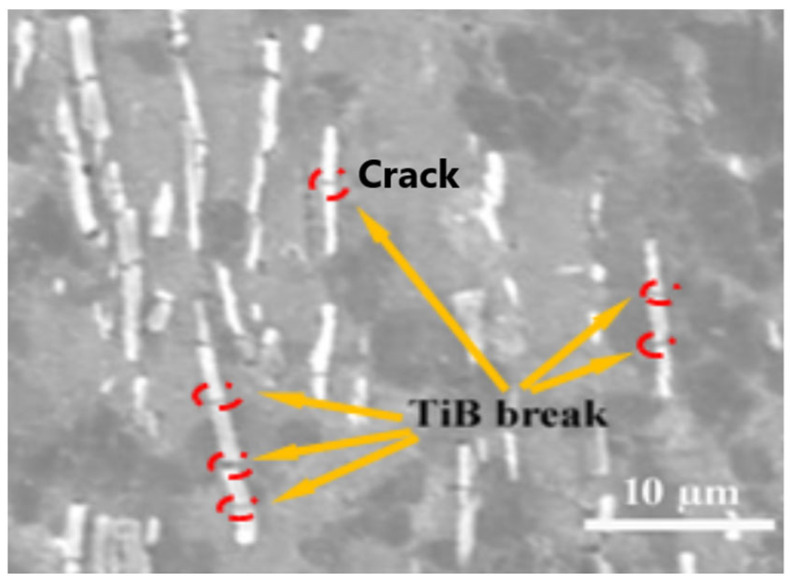
Breakage of TiB fibers during thermal processing.

**Figure 10:**
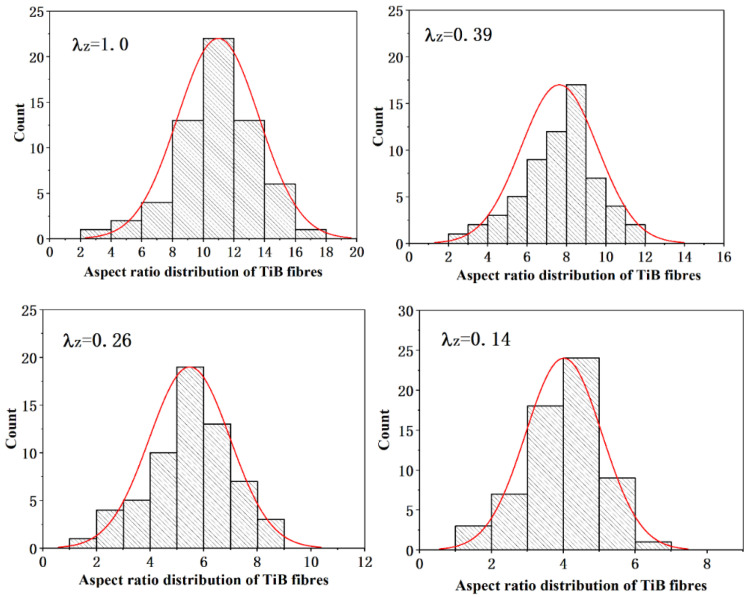
Changes in the aspect ratio of TiB fibers during thermal deformation.

However, as shown in Figure 9, broken TiB fibers were observed in the composite after thermal deformation, which decreased the aspect ratio of the TiB fibers. Figure 10 shows the changes in the aspect ratio of the TiB fibers after deformation to different extents. The results indicate that with an increase in the extent of deformation, breakage of the TiB fibers became severe, and even TiB fibers with a higher aspect ratio were broken several times by thermomechanical processing.

Huang et al. [40] investigated the breaking of TiB fibers during an extrusion process. Their results showed that the breakage of TiB fibers became severe with an increase in the extrusion angle. On the other hand, according to the stress transfer model described above, with an increase in the TiB fibers’ aspect ratio the TiB fibers may break, because the stress transferred to the TiB fibers is beyond their strength limit. According to the results and analysis above, the breakage of TiB during thermal deformation may result from a small deformation zone, a high deformation ratio or deformation rate, and the low temperature applied, which led to a sharp decrease in the aspect ratio of the fibers after thermal processing. It is widely understood that the breakage of TiB fibers during thermal deformation results from the stress exceeding the strength limit of TiB fibers. Below, we discuss how the breakage of TiB fibers is related to the relationship between the actual aspect ratio and the critical one.

### 3.3. Model of the Strengthening Effect of the Short In Situ TiB Fibers

The strengthening effect of TiB in the composite was modeled with a stress transfer model by the shear-lag method suggested by Cox [41]. In this model, the stress in the fibers of MMCs is transferred from the matrix, and only the shear stress is assumed to be transferred from the matrix to the fibers. The shear stress on the fiber–matrix interface was simplified to a constant. Equation (17) gives the stress in the fibers with an orientation parallel to the loading direction

(17) $$\sigma_{f}=\frac{\frac{1}{2}\pi d\tau_{i}l}{\frac{1}{4}\pi d^{2}}=\frac{2\tau_{i}l}{d}$$

where $\sigma_{f}$, l and d are the stress, length and diameter of the fibers, respectively; $\tau_{i}$ is the shear stress on the fiber–matrix interface. From this model, it can be deduced that the stress in the fibers or its strengthening effect is related to the interfacial shear stress and the aspect ratio of the fibers. The stress transferred to the fibers increases with the aspect ratio of the fibers and the shear stress on the interface. However, the fibers have a critical aspect ratio because the maximum shear stress on the interface is limited by the tensile strength of the fibers. Thus, on the basis of Equation (17), Equation (18) can be deduced

(18) $$AR_{c}=\frac{l_{c}}{d}=\frac{\sigma_{fu}}{2\tau_{y}}$$

where *ARc*, $l_{c}$ and d are the critical aspect ratio, the critical transferred length and the diameter of the fibers; $\tau_{y}$ refers to the shear strength of the fiber–matrix interface; and $\sigma_{fu}$ is the fibers’ tensile strength. Equation (19) gives the stress in the fibers [26]:

(19) $$\sigma_{f}=\left\{\begin{array}{c} \sigma_{fu}\left(1-\frac{l_{c}}{2l}\right),\left(l>l_{c}\right)\\ \sigma_{fu}\frac{l}{2l_{c}},\left(l<l_{c}\right) \end{array}\right.$$

**Figure 11:**
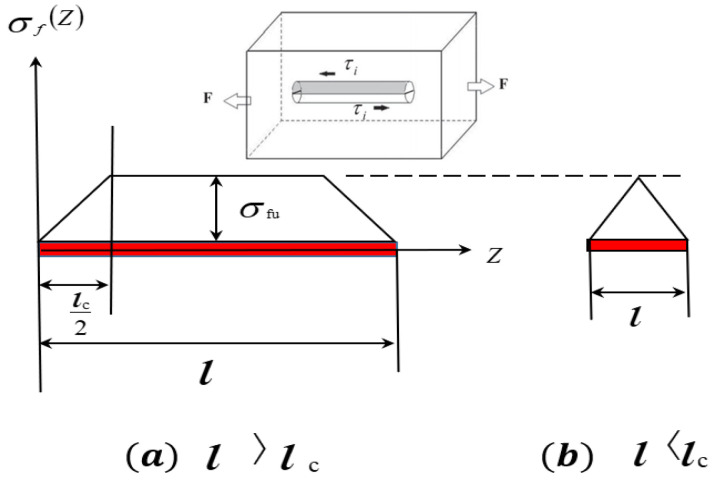
Stress as a function of the length/aspect ratio of TiB fibers (**a**) above the critical value and (**b**) below the critical value.

Figure 11 illustrates the stress as a function of the length/aspect ratio of TiB fibers with levels of stress above (Figure 11a) or below (Figure 11b) the critical level modeled with Equation (19). According to the model presented in Figure 11, the stress reaches the tensile strength limit of the TiB fibers with a length/aspect ratio greater than the critical one. Such TiB fibers tend to break during thermal deformation. TiB fibers with a length/aspect ratio significantly larger than the critical one may break several times with an increase in stress during tensile tests; the breakage of TiB during thermal deformation is shown in Figure 9. 

Because of the high shear strength of the TiB–matrix interface prepared by the in situ technique, the shear strength is assumed to be larger or equal to the shear strength of the titanium matrix. In other words, the maximum shear stress of the TiB–matrix composite is equal to the shear strength of the titanium matrix. The composite’s strength is given in Equation (20) [42]

(20) $$\sigma_{c}=\sigma_{f}V_{f}+\sigma_{m}\left(1-V_{f}\right)$$

where $\sigma_{c}$, $\sigma_{f},$ $\sigma_{m}$ and $V_{f}$ are the composite strength, the fibers’ strength, the strength of the matrix and the fibers’ volume fraction, respectively. In Equations (19) and (20), the orientation of the fibers is parallel to the loading direction. However, the orientation of the fibers synthesized in situ in this study was disordered. The effect of the fibers’ disorderly distribution on the strengthening effect is necessary to take into account in Equation (20).

For the case of fibers with variable lengths and a disordered orientation, Fukuda and Chou [43,44] suggested a probability density model to predict the strength of the composite. In their model, the strength of the composite with variable fiber lengths and a disordered fiber orientation was modified with a strengthening factor (*C*_0_) varying from 0 to 1. *C*_0_ is given by Equation (22)

(21) $$\sigma_{c}=\sigma_{f}V_{f}C_{0}+\sigma_{m}(1-V_{f})$$

(22) $$C_{0}=\int_{0}^{\frac{\pi }{2}}p\left(\theta \right)\cos\theta d\theta \int_{0}^{\theta_{0}}p\left(\theta \right)\cdot \cos^{3}\theta d\theta \int_{0}^{\theta_{0}}(1-\frac{\beta \;\bar{l}}{l\cos\theta })\cdot p\left(\theta \right)d\theta \left[\int_{\beta \;\bar{l}}^{l_{c}}\;\frac{l}{2l_{c}}h\left(l\right)\mathrm{dl}+\int_{l_{c}}^{\infty }(1-\frac{l_{c}}{2l})h\left(l\right)\mathrm{dl}\right]$$

where  θ  is the orientation angle, referring to the angle between the loading direction and the fibers’ longitudinal direction; p(θ) is a density function describing the probability of the fibers’ orientation;${\;\theta }_{0}$ is the critical value of θ; h(l) is a density function describing the probability of the fibers’ length; β is a constant showing whether a whisker is a bridging fiber or not; *l*, *lc* and $\;\bar{l}$ refer to the length, the critical length and the average length of the fibers, respectively. The critical aspect ratio of TiB fibers reported by Xiao et al. is ~2.07 in IMI834 [45]. In this research, the aspect ratio of the TiB fibers was significantly larger than its critical value; all fibers could be regarded as bridging fibers. The function *h(l)* was regarded as a constant, and *l* for all fibers was set to be larger than *lc* to simplify this model. Thus, β and ${\;\theta }_{0}$ in Equation (22) are 0 and $\frac{\pi }{2}$, respectively, and

(23) $$C_{0}=\int_{0}^{\frac{\pi }{2}}p\left(\theta \right)\cos\theta d\theta \int_{0}^{\frac{\pi }{2}}p\left(\theta \right)\cdot \cos^{3}\theta d\theta \int_{0}^{\frac{\pi }{2}}p\left(\theta \right)d\theta$$

From Equation (23), it can be seen that p(θ) should be taken first to calculate *C*_0_. As mentioned above, the TiB fibers in the as-cast composite were distributed randomly. In this case, $p\left(\theta \right)$ was deduced to be $p\left(\theta \right)=\sin\theta$.

By taking $p\left(\theta \right)=\sin\theta$ into Equation (23), we have

$$C_{0}=\int_{0}^{\frac{\pi }{2}}sin\theta \cdot cos\theta d\theta \int_{0}^{\frac{\pi }{2}}sin\theta \cdot cos^{3}\theta d\theta \int_{0}^{\frac{\pi }{2}}sin\theta d\theta =\frac{1}{8}\;\;$$

The calculated *C*_0_ of TiB fibers in the as-cast composite indicated that it was fairly small because of the random orientation distribution. As mentioned above, TiB fibers rotate toward the elongation direction during thermal deformation, as described by Equation (16). The evolution of the TiB fibers’ strengthening effect can be predicted with Equation (16) and Equation (23).

If we substitute Equation (16) into Equation (23), we have

(24) $$c_{0}=\int_{0}^{\frac{\pi }{2}}\frac{\sin\left(\arctan\left(\lambda^{-\frac{3}{2}}\tan\theta \right)\right)}{\lambda^{-\frac{3}{2}}\sin^{2}\theta +\lambda^{\frac{3}{2}}\cos^{2}\theta }\cdot \cos\theta d\theta \int_{0}^{\frac{\pi }{2}}\frac{\sin\left(\arctan\left(\lambda^{-\frac{3}{2}}\tan\theta \right)\right)}{\lambda^{-\frac{3}{2}}\sin^{2}\theta +\lambda^{\frac{3}{2}}\cos^{2}\theta }\cdot \cos^{3}\theta d\theta \int_{0}^{\frac{\pi }{2}}\frac{\sin\left(\arctan\left(\lambda^{-\frac{3}{2}}\tan\theta \right)\right)}{\lambda^{-\frac{3}{2}}\sin^{2}\theta +\lambda^{\frac{3}{2}}\cos^{2}\theta }d\theta \;$$

### 3.4. Strengthening through Microstructural Refinement

The strengthening effect resulting from microstructural refinement is evaluated with the Hall–Petch equation [46]

(25) $$\sigma_{\mathrm{ym}}=\sigma_{0}+K{\mathrm{Ged}}^{-\frac{1}{2}}$$

where $\sigma_{0},\;$*K* and Ged are the strength constant, the strengthening coefficient of grain refinement and the size of the equivalent grains, respectively. Our previous research showed that dislocations can slide through different alpha laths in the same colony because they have the same crystal orientation. However, the dislocations’ slippage is hindered effectively by the interface between the alpha colonies because of the different crystal orientations in them. The equivalent grain size of the composite is used as the average size of the alpha colonies [47]. The composite’s strength, taking the strengthening effect of the microstructure refinement into account in Equation (21), can be modified with Equation (26).

(26) $$\sigma_{c}=\sigma_{f}V_{f}C_{0}+(\sigma_{0}+K{\mathrm{Ged}}^{-\frac{1}{2}})\cdot (1-V_{f})$$

**Figure 12:**
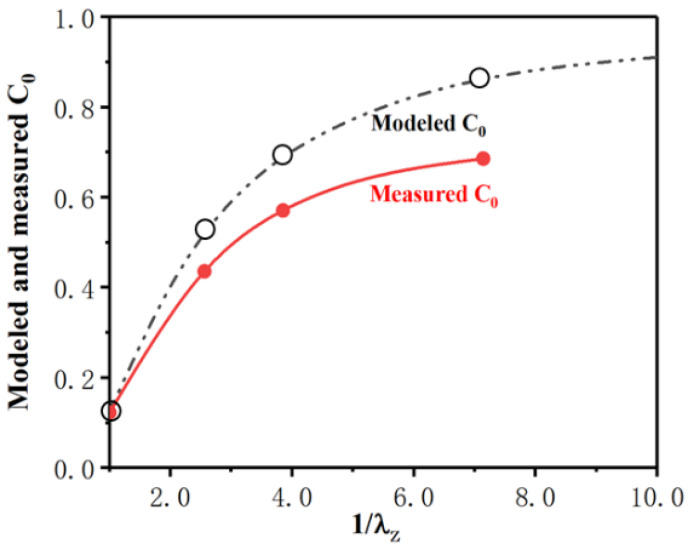
Modeled and measured strengthening factor (*C*_0_) of TiB fibers in the composite.

Table 1 lists the mechanical properties and the measured values of *C*_0_ of the composite after thermal processing with different deformation ratios. The values of *C*_0_ modeled by Equations (24) and (26) are also listed in Table 1. Figure 12 shows the changes in the measured and modeled *C*_0_ of TiB fibers with different deformation ratios. As shown in Figure 12, the modeled *C*_0_ of the TiB fibers agreed well with those measured in the as-cast composite. However, the modeled value deviates gradually from the measured one with an increase in the deformation ratio. The *C*_0_ of TiB fibers was overestimated by Equation (24). This overestimation may have resulted from the TiB fibers breaking during thermomechanical processing, as presented in Section 3.2.2. Because of the TiB fibers’ breakage during thermal processing, the average aspect ratio of the TiB fibers decreases with an increase in the extent of deformation, which results in less stress being transferred from the matrix and the smaller *C*_0_ of the TiB fibers.

## 4. Conclusions

A composite reinforced with TiB was prepared by the arc remelting and casting method by the authors. TiB was formed through the in situ synthesis reaction between titanium and boron, then grown into fibers.

1. The orientation of the TiB fibers was totally random in the as-cast composite, and the probability density distribution function of the TiB fibers’ orientation (P(θ)) with respect to the direction of elongation can be described as sinθ. Based on a shear-lag model of stress transfer, the equation of the strengthening factor (*C*_0_) of in situ TiB fibers was simplified. In this equation, *C*_0_ can be calculated with the distribution density function of the TiB fibers’ orientation (P(θ)), and *C*_0_ was calculated to be just 0.125 for the as-cast composite because of the random distribution of the TiB fibers’ orientation.
2. The distribution of the aspect ratio of the TiB fibers was changed by thermal processing because the TiB fibers broke. The aspect ratio of the fibers decreased with the deformation ratio, which could be interpreted by the shear-lag model. According to this model, TiB fibers tend to break if their length/aspect ratio is larger than the critical length/aspect ratio. Furthermore, the alpha colonies in the composite were refined significantly by thermal deformation.
3. With the pseudo-affine deformation scheme, the evolution of the TiB fibers’ orientation and their probability density distribution function during compression deformation was deduced and modeled. With this model, the strengthening factor (*C*_0_) was calculated to be 0.520, 0.688 and 0.858 for λ_Z_ = 0.39, 0.26 and 0.14, respectively. Compared with the as-cast composite, *C*_0_ was improved significantly by thermal processing with different deformation ratios. The modeled *C*_0_ of the TiB fibers agreed well with the measured *C*_0_ of the as-cast composite. However, the modeled values deviated gradually upward from the measured ones when the deformation ratio increased. This deviation in *C*_0_ may be attributed to the TiB fibers breaking during thermomechanical processing, which decreased the stress in the TiB fibers transferred from the matrix described by the shear-lag model.

## Figures and Tables

**Table 1:** Mechanical properties and the *C*_0_ values of the composite after thermal processing with different levels of deformation.

Deformation Ratio (λ)		UTS (MPa) 823 K	δ (%) 823 K	UTS (MPa) 923 K	δ (%) 923 K	Modeled C_0_	Measured C_0_	Relative Error of C_0_ (%)
λx, λy	λz							
1.00	1.00	620.2 ± 41	7.3 ± 0.4	582.3 ± 33	7.9 ± 0.7	0.125	0.123	−1.626
1.56	0.39	713.8 ± 46	10.6 ± 0.8	657.5 ± 41	11.7 ± 0.9	0.520	0.488	−6.557
1.96	0.26	789.6 ± 56	14.5 ± 0.8	719.3 ± 45	16.3 ± 1.1	0.688	0.623	−10.433
2.67	0.14	819.3 ± 62	15.3 ± 1.0	742.7 ± 53	18.1 ± 1.2	0.858	0.729	−17.695
